# Sensing mechanisms of iron–sulfur cluster regulatory proteins elucidated using native mass spectrometry

**DOI:** 10.1039/d1dt00993a

**Published:** 2021-05-17

**Authors:** Jason C. Crack, Elizabeth Gray, Nick E. Le Brun

**Affiliations:** Centre for Molecular and Structural Biochemistry, School of Chemistry, University of East Anglia, Norwich Research Park Norwich NR4 7TJ UK j.crack@uea.ac.uk n.le-brun@uea.ac.uk

## Abstract

The ability to sense and respond to various key environmental cues is important for the survival and adaptability of many bacteria, including pathogens. The particular sensitivity of iron–sulfur (Fe–S) clusters is exploited in nature, such that multiple sensor-regulator proteins, which coordinate the detection of analytes with a (in many cases) global transcriptional response, are Fe–S cluster proteins. The fragility and sensitivity of these Fe–S clusters make studying such proteins difficult, and gaining insight of what they sense, and how they sense it and transduce the signal to affect transcription, is a major challenge. While mass spectrometry is very widely used in biological research, it is normally employed under denaturing conditions where non-covalently attached cofactors are lost. However, mass spectrometry under conditions where the protein retains its native structure and, thus, cofactors, is now itself a flourishing field, and the application of such ‘native’ mass spectrometry to study metalloproteins is now relatively widespread. Here we describe recent advances in using native MS to study Fe–S cluster proteins. Through its ability to accurately measure mass changes that reflect chemistry occurring at the cluster, this approach has yielded a remarkable richness of information that is not accessible by other, more traditional techniques.

## Abstract 2

Native mass spectrometry provides novel insight into protein cofactor (*e.g.* iron–sulfur cluster) chemistry.

## Graphical abstract

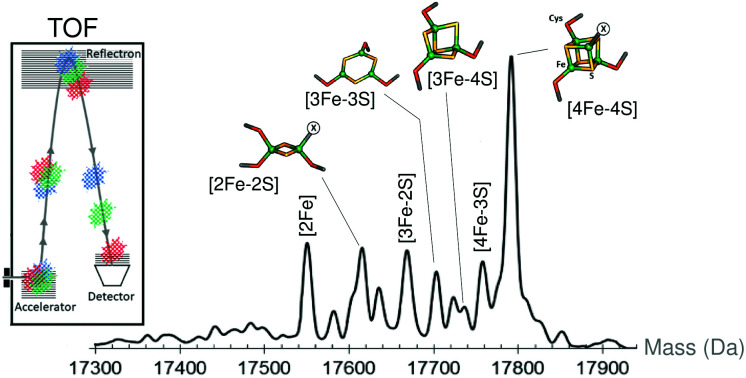

## 1. Introduction

Electrospray ionization mass spectrometry (ESI-MS), coupled with liquid chromatography (LC), is today's method of choice for proteomic analysis. However, proteins rarely retain their *native* structure in the solvent mixtures (*e.g.* water/acetonitrile/formic acid) employed in such experiments, leading to a loss of non-covalent interactions. In the early 90s, the observation of non-covalently bound heme in myoglobin samples suggested ESI-MS could be used to study non-covalent protein–cofactor interactions.^1^ This fledgling field of non-denaturing ESI-MS (for a contemporary review see ref. 2) was set to capitalise on the spectacular advances in both mass spectrometric hardware (particularly in resolution and sensitivity) through the 90s to the present day. In 2004 the term *native* MS was introduced to unify the bewildering terminology (*e.g.*, non-denaturing, non-covalent, native spray, macro- or supra-molecular ESI-MS) that was being used to describe similar approaches to ESI-MS experiments.^3,4^ Almost two decades later, native MS is now used for accurate mass detection of intact proteins and protein complexes, and has been used extensively to study the dynamics of protein–protein interactions, and interactions of proteins with drugs, nucleic acids, sugars, lipids and metals.^5–15^

**Fig. 1:**
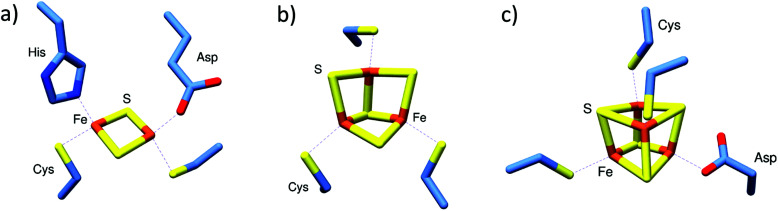
Commonly found iron–sulfur cluster protein cofactors. (a) [2Fe–2S] cluster of *Streptomyces venezuelae* RsrR with unique coordination by Cys (×2), His and Asp residues (PDB 6HSD^22^). (b) All Cys-coordinated [3Fe–4S] cluster of *E. coli* aconitase (PDB 1L5J^23^). (c) [4Fe–4S] cluster of *Streptomyces coelicolor* NsrR, coordinated by Cys (3) and Asp (PDB 5N07^24^). Images generated using UCSF Chimera.^25^

It is estimated that up to a half of all proteins utilise a metal ion for structural stability and/or function.^16^ The greatest diversity of functional properties is exhibited by ions of the transition metal elements (principally first row), of which iron is the most abundant in biology. Iron occurs in three principal forms in life: as heme (iron coordinated within the centre of a porphyrin macrocycle); as non-heme iron (iron coordinated directly to proteins *via* amino acid side chains); and, as iron–sulfur (Fe–S) clusters, ancient protein cofactors composed solely of iron and inorganic sulfide, which are bound to proteins *via* the coordination of iron by amino acid side chains,^17–19^ see Fig. 1. Fe–S cluster proteins are widespread in nature and exhibit a remarkable breadth of structural and function diversity. Many play key roles in electron transfer, chemical catalysis and small molecule sensing.^20,21^

Mass spectrometry approaches have been instrumental in the identification and characterisation of a wide variety of metalloproteins.^26–36^ Fe–S clusters have been generally less amenable to investigation by mass spectrometry because of their sensitivity; they are acid-labile and were invariably lost under the mildly acidic conditions employed during early ESI-MS investigations, with only the most robust Fe–S proteins retaining their cluster following ionization. In one of the earliest examples, the high potential Fe–S protein (HiPIP) from *C. tepidum* was found to exhibit three distinct charge states (+5 to +7), reminiscent of the *new* charge states observed a few years earlier for heme containing myoglobin.^1,37^ Deconvolution of the HiPIP spectrum was consistent with molecular mass of the protein complete with [4Fe–4S] cluster.^37^ HiPIP proteins belong to the broader ferredoxin family, members of which naturally display high degree of stability at or near neutral pH. Thus, they were the obvious choice to illustrate the potential of mass spectrometric approaches, and to help define a set of conditions suitable for the detection of other less robust Fe–S proteins. In most cases, the Fe–S cluster(s) survived ionization and remained associated with the protein irrespective of whether they were [2Fe–2S] or [4Fe–4S] cluster.^38–41^ Recently, native MS has been applied to a much broader range of Fe–S proteins, enabling the determination of cluster types, and, remarkably, the nature of chemistry taking place at clusters, in some cases in real time.^42–52^ Aspects of these advances are the focus of this article.

## 2. Native MS of Fe–S proteins reveals the type of cluster

The presence of an Fe–S cluster in purified protein samples is most commonly assessed using UV-visible absorption spectroscopy, with the spectra of [4Fe–4S] and [2Fe–2S] clusters being sufficiently distinct to give an indication of the cluster type.^53^ However, additional, more definitive evidence is required for unambiguous assignment. Recently, native MS has proved extremely useful for determining the type of Fe–S cluster ligated to the protein framework. In comparison to other techniques, such as electron paramagnetic resonance (EPR), Mössbauer, and resonance Raman spectroscopies, native MS requires significantly less sample (≤20 μM), can simultaneously resolve all Fe–S cluster species present in the sample, does not require a specific oxidation state for detection, and does not typically require isotopic enrichment.^44–46,54,55^

Native MS has been widely used in determining the cluster type of members of the Rrf2 family of bacterial transcriptional regulators, which function in sensing a wide range of environmentally important cues: where characterized, members of this family have been found to sense nitric oxide (NO)/nitrosative stress (NsrR),^43,56^ redox balance/oxidative stress (HypR, SaiR, RsrR),^54,57,58^ cysteine availability (CymR),^59^ the iron–sulfur cluster status of the cell (IscR),^60–62^ and iron limitation (RirA).^45,48,63,64^ Members of the family can be broadly divided according to whether or not they utilise an Fe–S cluster as the sensory module.^59,65^ Here we highlight the use of native MS in determining the type of Fe–S cluster bound to the protein framework of three Rrf2 members: NsrR, RsrR and RirA.

### 2.1 [4Fe–4S] NsrR

NsrR functions as a regulator of NO-induced stress response in many bacterial species.^43,56^ In some bacteria, such as *E. coli* and *S. typhimurium*, ≥60 genes are under NsrR control, while in others, such as *Neisseria* sp. and *Streptomyces coelicolor*, a more compact (≤20) set of regulated genes is observed.^42,66–68^ However, many of the core genes controlled by NsrR function in the detoxification of NO by converting it to non-toxic metabolites. The principal target in most species is the *hmp* gene, which encodes a flavohaemoglobin that converts NO to nitrate under aerobic conditions.^67,69^

Initial aerobic purifications of *S. coelicolor* NsrR resulted in the presence of a [2Fe–2S] cluster (based on absorbance spectroscopy), while later anaerobic preparations resulted in protein containing a [4Fe–4S] cluster, or occasionally a mixture of [4Fe–4S] and [2Fe–2S] clusters.^70,71^ Although initially unclear, the relationship between [4Fe–4S] NsrR and the previously reported [2Fe–2S] form was revealed by native MS to be dependent upon the presence of *protective* low molecular weight thiols added during the initial purifications.^42^ The presence of these thiols (*e.g.* β-mercaptoethanol and other non-physiological small thiols) under aerobic conditions promoted the conversion of the native [4Fe–4S] cluster to a stabilised [2Fe–2S] form. The recent high resolution X-ray structure of NsrR confirmed the nature of the [4Fe–4S] cluster, and revealed a unique coordination of the cluster at the interface of the two subunits of the dimer, by three Cys residues of one subunit and an Asp residue of the other (Fig. 1c).^24^

### 2.2 [2Fe–2S] RsrR

*S. venezuelae* RsrR was originally annotated as a putative homolog of NsrR, but the nature of its regulon indicated that it is involved in sensing redox balance (*e.g.* it regulates *nmrA*, which encodes a NAD(P)-dependent transcriptional regulator and several genes associated with mycothiol synthesis).^54^ In contrast to NsrR, anaerobic preparations of RsrR were pink in colour with a series of weak absorptions bands across the 300–600 nm region reminiscent of some [2Fe–2S] clusters with non-Cys coordination. The mass spectrum contained peaks in regions that corresponded to monomeric and dimeric forms of [2Fe–2S] RsrR.^54^ The partial dissociation of proteins that are dimeric in solution into monomers is a well-known phenomenon. This may result from a number of causes, including a simple reflection of a solution equilibrium between the two forms, an excess of energy applied following transfer to the gas phase (collisional activation), or the disruption of hydrophobic interactions that stabilise the dimer during transfer to the gas phase.^72^ Whatever its origin, this has proved to be advantageous because it simplifies the assignment of Fe–S species, which could potentially be different in the two subunits of a dimeric Fe–S protein.^44,48^ The nature of RsrR cluster was confirmed by the high resolution X-ray structure, which revealed unique cluster coordination involving two Cys, one Glu and one His residues, the first example of a cluster coordinated by three different amino acid residues, and the first [2Fe–2S] cluster characterised with Glu coordination (Fig. 1a).^22^

### 2.3 [4Fe–4S] RirA

RirA was discovered first in *Rhizobium leguminosarum*, the nitrogen-fixing symbiont that induces root nodules on peas, and is also found in several closely related genera of α-proteobacteria, including other Rhizobia and other α-proteobacteria, including the pathogens *Bartonella*, *Brucella*, and *Agrobacterium.*^63,73^ It represses many genes involved in iron homeostasis (*e.g.* siderophore biosynthesis, and heme uptake) by binding to operator sequences known as “IRO boxes”. RirA is a member of the Rrf2 family and was hypothesised to be an Fe–S protein almost 15 years ago.^74^

**Fig. 2:**
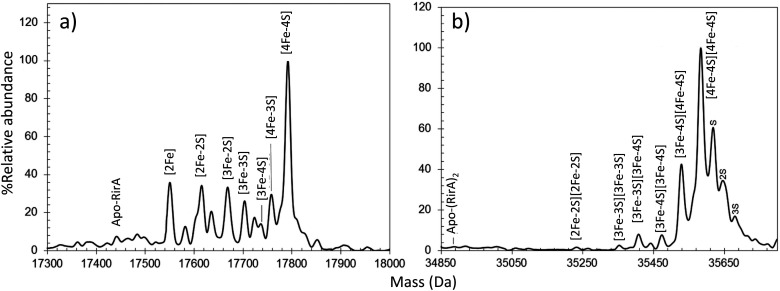
Characterization of the RirA Fe–S cluster by native MS. Deconvoluted native MS spectrum of [4Fe–4S] RirA in the (a) monomeric and (b) dimeric regions. See ref. 45 for further details.

Anaerobic preparations of RirA resulted in the presence of a [4Fe–4S] cluster (based on absorbance), which had an enhanced [3Fe–4S]^1+^ EPR signal following anaerobic gel filtration. This behaviour suggested the cluster has a labile fourth iron. Again, both monomeric and dimeric forms of the protein were observed by native MS^45^ (Fig. 2). A dominant peak at 17 792 Da, corresponding to [4Fe–4S] RirA, was observed in the monomer region. Minor, well resolved peaks were observed on the low mass side, correspond to traces of [4Fe–3S], [3Fe–4S], [3Fe–3S], [3Fe–2S] and [2Fe–2S] cluster forms (Fig. 2a). Homo-dimeric [4Fe–4S] RirA dominated the dimer region of the spectrum at 35 585 Da. Dimers containing a mixture of [3Fe–4S] and [4Fe–4S] clusters, were also observed.^45^ The observation of [3Fe–4S] clusters following preparation for native MS, which involves gel filtration, was consistent with EPR observations (Fig. 2b).^45,75^ The physiological significance of [4Fe–4S] RirA was strongly supported by the demonstration that this form of the protein binds IRO box sequences. Exposure to low iron conditions initiates loss of iron to initially generate a [2Fe–2S] form, which exhibits much weaker DNA-binding affinity before degrading further to apo-RirA, which does not bind DNA.^45^

## 3. Time-resolved native MS reveals the sensing mechanism of regulatory Fe–S proteins

For processes that involve changes in mass at the active site of a protein/enzyme, mass spectrometry has the potential to reveal precise detail of the chemistry taking place, and in some favourable cases this can be followed in real time. Studies of FNR and RirA, described below, illustrate the power of time-resolved native mass spectrometry.

### 3.1 [4Fe–4S] FNR

*Escherichia coli* and many other organisms use FNR as a sensor of O_2_ levels, enabling it to switch between aerobic and anaerobic metabolism according to O_2_ availability. The Fe–S cluster of FNR is key to the O_2_ sensing mechanism: in the absence of O_2_, the protein binds a [4Fe–4S] cluster, is homodimeric and binds tightly to FNR operator sequences located in FNR-controlled promoter regions, either activating or repressing transcription. Through a combination of, Mössbauer, EPR and optical spectroscopies, the [4Fe–4S] cluster of *E. coli* FNR was shown to undergo a [4Fe–4S] to [2Fe–2S] cluster conversion in response to O_2_,^76–79^ and that this involves a transient [3Fe–4S] intermediate.^80–82^ The [2Fe–2S] form of FNR is monomeric and can no longer bind DNA with high affinity.^83^ The cluster conversion reaction provides the driving force for the conformational change required to disrupt dimerisation/DNA binding.

The nature of the conversion process was revealed to be more complex than originally thought with the discovery of persulfide-ligated [2Fe–2S] clusters by resonance Raman spectroscopy.^84^ Native MS was subsequently used to investigate the origin of these persulfide-ligated [2Fe–2S] clusters in a site-directed variant of FNR, S24F (hereafter referred to as FNR), that was chosen because it is spectroscopically identical to wild type FNR, follows the sample [4Fe–4S] to [2Fe–2S] cluster conversion, but does so significantly more slowly,^44,85^ thus enabling the application of time-resolved native MS.

**Fig. 3:**
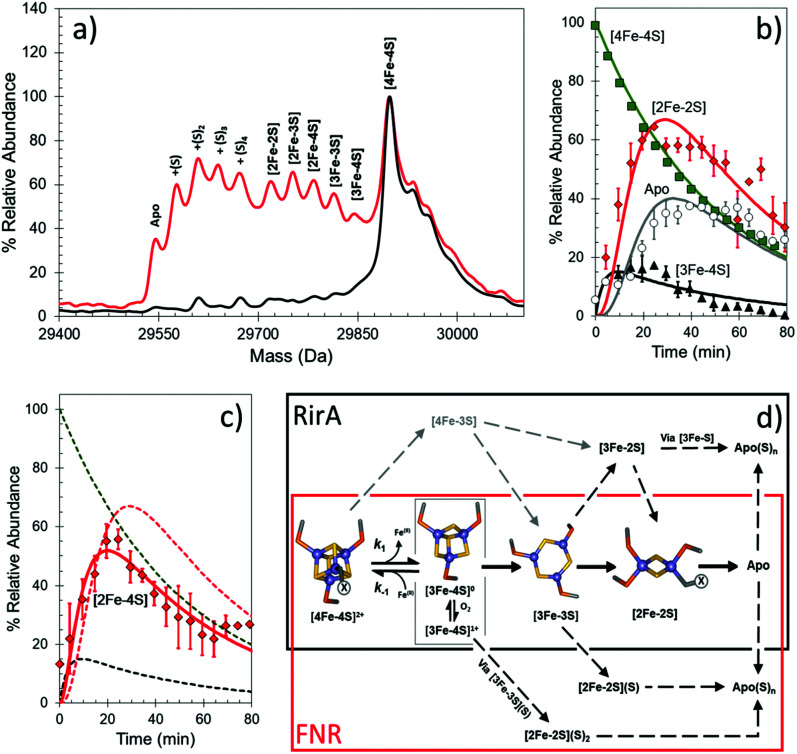
Native MS of [4Fe–4S] FNR and the effect of O_2_ exposure. (a) Deconvoluted mass spectrum of [4Fe–4S] S24F FNR before (black line) and after exposure (18 min, red line) to dissolved O_2_. This resulted in the formation of a variety of protein-bound clusters including [3Fe–4S], [3Fe–3S] and [2Fe–2S] forms. Persulfide adducts of the [2Fe–2S] cluster and apo-FNR were also observed. (b) and (c) Temporal behaviours of the [4Fe–4S] cluster (black squares), [3Fe–4S] cluster (yellow triangles), [2Fe–2S] cluster (red diamonds) and apo FNR (white circles) forms (in b), and [2Fe–4S] cluster form (red diamonds) in (c) after O_2_ exposure. Global fitting to the experimental data is shown as solid lines. See ref. 44 for further details. (d) Proposed mechanistic scheme for the conversion of [4Fe–4S] FNR and RirA based on ESI-MS data.^44,45^ The initial [4Fe–4S] cluster is coordinated by three Cys residues. The fourth ligand, illustrated in the figure as “X”, is a Cys in FNR, but unknown for RirA. Both regulators follow a common [4Fe–4S] to [2Fe–2S] conversion pathway, *via* [3Fe–4S] and [3Fe–3S] clusters. The two mechanisms diverge at indicated branch points to give persulfide ligated [2Fe–2S] clusters (FNR, red box) or other FeS cluster types (RirA, black box).

Under anaerobic conditions, the monomer region of the mass spectrum featured a major peak at 29 892 Da, while a peak at 59 796 Da dominated the dimeric region. These observations are consistent with the presence of [4Fe–4S] FNR. Exposure to O_2_ resulted in the formation of a variety of protein-bound clusters, including the previously observed [3Fe–4S] (29 843 Da) and [2Fe–2S] (29 720 Da) forms, along with an unexpected [3Fe–3S] cluster (29 811 Da).^44^ Single (29 752 Da) and double (29 784 Da) persulfide ligated forms of [2Fe–2S] cluster were also observed, along with persulfide adducts of apo FNR (Fig. 3a).

The data also revealed the temporal behaviour for each observed species (Fig. 3b and c). In the dimeric region, aside from the [4Fe–4S]/[4Fe–4S] peak, only three very low intensity peaks were observed following reaction with O_2_, each corresponding to an FNR dimer containing at least one [3Fe–4S] cluster. This is consistent with cluster conversion initiating the FNR dimer to monomer transition that modulates DNA binding.^44,78^ Global analysis of multiple data sets led to the development of a model that describes the kinetic characteristics of the main [4Fe–4S], [3Fe–4S] and [2Fe–2S] cluster species, consistent with previously solution-based studies, as well as those of the newly discovered [3Fe–3S] and persulfide-bound [2Fe–2S] forms. This study illustrated the remarkable power of native MS to reveal intricate mechanistic details (Fig. 3d).^44^

**Fig. 4:**
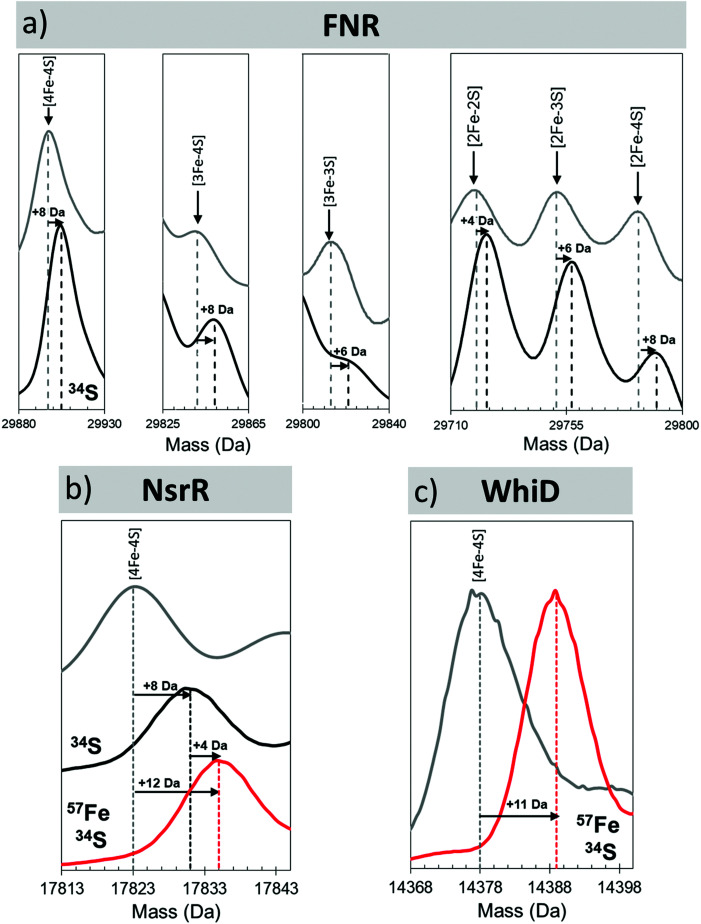
Mass shifts of Fe–S species due to ^34^S and/or ^57^Fe substitution. Deconvoluted mass spectra of natural abundance and isotopically labelled clusters and intermediates from (a) FNR, (b) NsrR and (c) WhiD. Cluster species and isotope enrichment as indicated.^44,47^

The unambiguous identification of Fe–S cluster intermediates benefits significantly from the use of heavier isotopes of iron and/or sulfur. For the latter, ^34^S has been used, and methods optimised for the specific replacement of cluster sulfide (with protein sulfur remaining at natural abundance). This results in a +2 Da mass shift for each sulfide, see Fig. 4.

### 3.2 [4Fe–4S] RirA

**Fig. 5:**
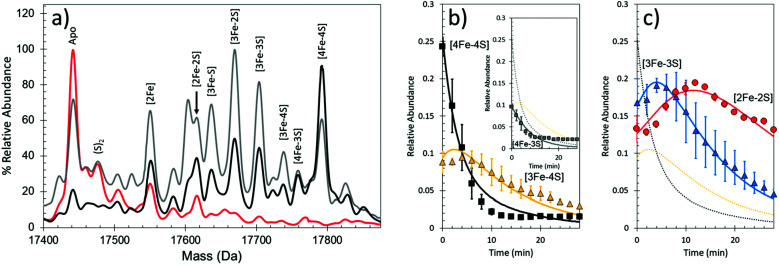
Native MS of [4Fe–4S] RirA in response to low iron conditions. (a) Time dependent changes in anaerobic [4Fe–4S] RirA treated with EDTA to simulate low iron conditions. Spectra were recorded at 0 min (black line), 4 min (grey line) and 30 min (red line) post exposure to EDTA. (b) and (c) Temporal behaviours of the [4Fe–4S] (black squares), [3Fe–4S] (yellow triangles) (b), [3Fe–3S] (blue triangles) and [2Fe–2S] clusters (red circles) (c). Global fitting to the experimental data is shown as solid lines. See ref. 48 for further details.

Under low iron conditions (induced by the iron chelator EDTA), [4Fe–4S] RirA was found to undergo a cluster conversion reaction resulting in a form with absorbance features reminiscent of a [2Fe–2S] cluster. The initial native MS spectrum of RirA (Fig. 5), containing an array of well resolved Fe–S cluster species, suggested it might be possible to follow cluster conversion of both the monomeric and dimeric forms.^45^ Under low iron and anaerobic conditions the [4Fe–4S] RirA peak at 17 792 Da in the monomeric region was observed to decay away, and new peaks corresponding to protein bound cluster fragments, including [4Fe–3S], [3Fe–4S], [3Fe–3S], [3Fe–2S], [3Fe–S], [2Fe–2S] and [2Fe–S] (17 586–17 762 Da) initially increased in intensity before eventually decaying away to apo-RirA (Fig. 5). The same set of intermediates were also observed under aerobic conditions, but the kinetics of their formation and loss were different; see ref. 48.

To determine the sequence of events, the temporal behaviour of each of species was investigated. The data showed that the [4Fe–3S] and [3Fe–4S] species were the first intermediates to reach their maximum abundance, consistent with them being early intermediates in the conversion process. The [3Fe–3S] species was the next intermediate to maximize, followed by the [3Fe–2S] and [2Fe–2S] forms.^48^

Global analysis of anaerobic data indicated that the loss of a single iron ion was the obligatory first step in the conversion process (formation of the [4Fe–3S] cluster form was minor and most likely resulted from damage to the cluster during ionization). The temporal behaviour of the [3Fe–3S] species was consistent with it being an intermediate on the [4Fe–4S] to [2Fe–2S] cluster conversion pathway (Fig. 5b and c). The model also indicates that the [3Fe–3S] species may decay to a [3Fe–2S] species, on route to apo-RirA (in part *via* a [2Fe–2S] species) (Fig. 3d, black box). While a [2Fe–S] species was observed, its temporal behaviour could not be sensibly modelled, suggesting that it might, at least in part, arise from the spontaneous re-assembly of iron/sulfide species released during the cluster conversion process.^48^

The formation/decay of several intermediates was accelerated in the presence of O_2_. However, the rate constant for the initial [4Fe–4S] to [3Fe–4S] conversion, *k* = 0.32 min^−1^, was independent of O_2_ (as found from solution studies), indicating that the loss of and an Fe ion from the [4Fe–4S] cluster is the rate limiting step of the conversion process.^45,48^ The led to the proposal that this is the iron sensing reaction of RirA. In the dimeric region, multiple peaks were observed primarily corresponding to RirA dimers containing a pair of [4Fe–4S], [3Fe–4S], or [2Fe–2S] cluster. The global analysis model developed for the monomeric form of RirA was broadly applicable to the more physiologically relevant dimeric species.^48^

## 4. Reaction of nitric oxide with Fe–S regulatory proteins followed by native MS

The susceptibility of Fe–S clusters to chemical reaction with reactive nitrogen species, principally NO, is well known, and is recognised as one of the main routes of NO toxicity.^83,86^ Such reactivity has been exploited through the evolution of Fe–S cluster regulatory proteins that control the cell's response to nitrosative stress. Studies of several [4Fe–4S]-containing proteins revealed a rapid reaction with 8–10 NO per cluster, resulting in the formation of iron–nitrosyl species similar in nature to the well characterised low molecular weight dinitrosyl iron complexes (DNIC), Roussin's Red Ester (RRE) and Roussin's Black Salt (RBS).^42,43,87,88^ In general, only a small proportion of these were of the DNIC type, which can be readily detected by EPR spectroscopy, with the majority of the RRE- and RBS-types, which are diamagnetic and harder to characterise. Thus, identifying the precise nature of these products, and the mechanisms by which they are generated, has been extremely challenging. The recent application of native MS has resulted in significant progress in understanding mechanisms of sensing.

### 4.1 [4Fe–4S] NsrR

NsrR functions as a regulator of the response to reactive nitrogen species (RNS), including nitric oxide (NO), in many bacterial species.^42,68^ In its [4Fe–4S] form, NsrR is able to bind DNA and thus repress the cell's response to NO stress. Upon exposure to NO, the cluster undergoes a rapid, complex, nitrosylation reaction resulting in the loss of DNA-binding (at ≥2 NO per cluster) and the formation of a mixture of nitrosyl species of the DNIC, RRE and RBS.^42,43,88^ Recent application of LC-ESI-MS enabled the first unambiguous identification of NsrR-bound RRE-type species, including a persulfide-bound form.^89^

Identification of intermediates of cluster nitrosylation is particularly challenging due the rapid rate of reaction, the transient nature of the intermediates and the spectroscopic similarity between iron–nitrosyl species.^86–88,90–93^ To try to address this, a slow NO-releasing reagent, dipropylenetriamine (DPTA) NONOate, was chosen as the NO source for native MS experiments. Under these conditions, NO availability limits the reaction, enabling an *in situ* NO titration of samples during native MS data acquisition.^47^

**Fig. 6:**
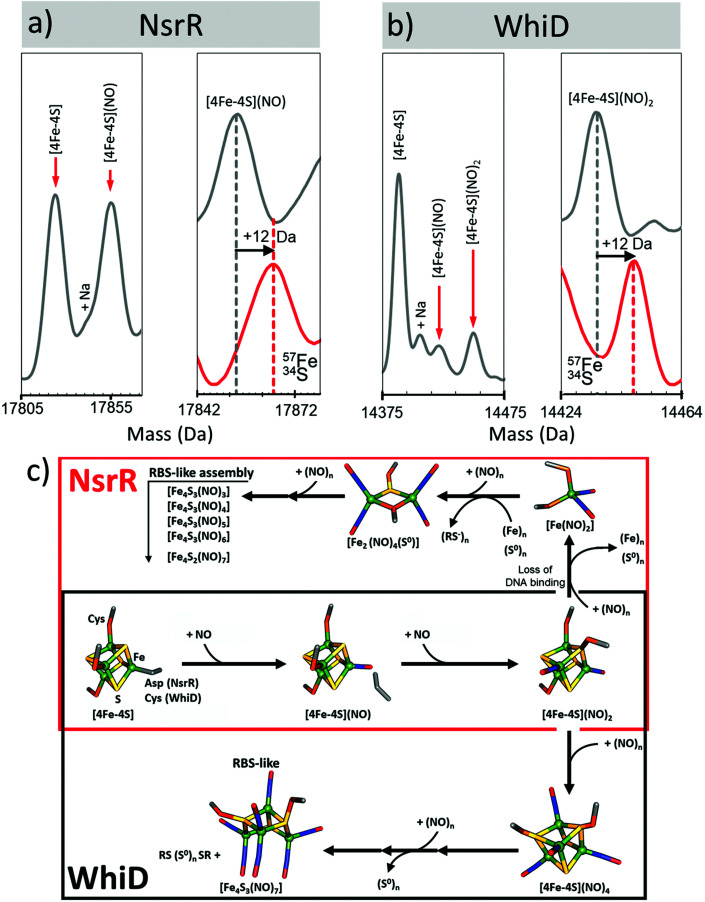
Proposed mechanistic scheme for the nitrosylation of [4Fe–4S] NsrR and WhiD based on native MS data. Earliest nitrosylation intermediates for (a) NsrR and (b) WhiD along with mass shifts due to isotopic enrichment. (c) Proposed mechanistic scheme for the nitrosylation of [4Fe–4S] NsrR and WhiD based on ESI-MS data. Starting from the [4Fe–4S] cluster both regulators (red box, NsrR; black box, WhiD) follow the same initial path, *via* mono- and dinitrosyl complexes (over lapping boxes). The two mechanisms then diverge. The NsrR cluster breaks down resulting in a loss of DNA binding. DNIC, RRE and RBS-like species then begin to increase in abundance. For WhiD, a [4Fe–4S](NO)_4_ species is formed on route to an RBS-like species. The structures illustrated here for [4Fe–4S](NO)_2_, [4Fe–4S](NO)_4_, [Fe_4_S_3_(NO)_7_] are speculative, and represent chemically reasonable interpretations for the events occurring. The structure of the RBS-like species is based on other spectroscopic data. See ref. 47 for further details.

In the presence of NO, the peak due to [4Fe–4S] NsrR decayed away with the concomitant formation of a new peak at 17 854 Da (+30 Da), consistent with the addition of a single NO molecule to the [4Fe–4S] cluster (Fig. 6a). The [4Fe–4S](NO) peak initially increased in intensity, before decaying away at [NO] : [4Fe–4S] ratios ≥3. A second species of NsrR, +60 Da heavier and very likely corresponding to [4Fe–4S](NO)_2_, was also observed to increase in intensity as NO was added, such that by [NO] : [4Fe–4S] ≈ 4, it was at least as intense as the +30 Da adduct. However, an unambiguous assignment could not be made for this +60 Da adduct, even with the use of ^34^S and ^57^Fe isotopes, due to the presence of other competing adducts (typically Na) that obscured its detection.

A range of other iron–nitrosyl species were also detected at higher (≥4) levels of NO. These occurred in the mass regions that correspond to DNICs (17 588 Da, [Fe(NO)_2_(Cys)_2_]), RRE and RBS-like protein adducts. The formation of DNICs began almost immediately following the introduction of NO, but with the majority of the intensity being observed at [NO] : [4Fe–4S] ratios ≥6, indicating that DNICs are a primary product of the nitrosylation reaction.^47^

In the RRE region, a persulfide coordinated species (17 736 Da, [Fe_2_(NO)_4_(S)]) increased in abundance markedly at [NO] : [4Fe–4S] ≥ 6, reaching a maximum [NO] : [4Fe–4S] ≈ 9, consistent with earlier LC-ESI-MS data.^89^ Multiple RBS-like species were also detected with general formula [Fe_4_(S)_3_(NO)_*x*_], where *x* = 3 to 6. It was noteworthy that RBS itself, [Fe_4_(S)_3_(NO)_7_], was not observed,^47,94^ though a putative intermediate in the conversion of RRE- to RBS, [Fe_4_(S)_2_(NO)_7_], was.^47,95^ All RRE- and RBS-like species exhibited a similar intensity profile, increasing above [NO] : [4Fe–4S] ≥ 6, suggesting the interconversion of RRE and RBS species, or breakdown of higher mass nitrosyl species, see Fig. 6c.^47,94^ The concomitant increase in the intensity of all apo-NsrR species at equivalent ratios suggested the protein-bound products of nitrosylation become unstable at high [NO] : [4Fe–4S] ratios.^47^

### 4.2 [4Fe–4S] WhiD

In contrast to NsrR, the WhiB-like (Wbl) family of regulators (named after the first discovered WhiB protein) are, remarkably, found only in the actinobacteria, a phylum of Gram-positive bacteria that includes *Streptomyces*, the most abundant source of clinically important antibiotics, and important pathogens such as *Mycobacterium tuberculosis* and *Corynebacterium diphtheriae*. Wbl proteins are generally small (∼10–15 kDa) and contain a highly conserved pattern of Cys residues C(*x*_*n*_)C(*x*_2_)C(*x*_5_)C that will bind an Fe–S cluster.^96^

In *S. coelicolor*, WhiB is required for the initiation of sporulation septation, and WhiD is required for the late stages of sporulation.^97^ In *M. tuberculosis*, Wbl proteins are required for the ability of the pathogen to persist within its host for long periods, as well as its remarkable tolerance to a wide range of antibiotics.^98^*M. tuberculosis* WhiB3, which is the mycobacterial homologue of *S. coelicolor* WhiD, contributes to virulence and is induced in mouse lungs and macrophages. It regulates lipid and polyketide biosynthesis, including tri-acylglycerol accumulation, *in vivo*, in response to activated macrophages.^99^ [4Fe–4S] WhiB3 was shown to react with NO, leading to the suggestion that WhiB3 acts as a sensor of O_2_ and NO to control expression of genes involved in intermediary metabolism.^100^ This provided a key connection to the well documented accumulation in *M. tuberculosis* of tri-acylglycerol (which is also present in the sputum of tuberculosis patients) in response to hypoxia and NO exposure.^101^*M. tuberculosis* WhiB1 is essential for cell viability and has been shown to bind specific DNA sequences following cluster nitrosylation,^102^ and to bind the principal housekeeping Sigma factor of the cell in a cluster-dependent manner.^46^

Native MS revealed that, like NsrR, the addition of NO led to the loss of the [4Fe–4S] WhiD peak. However, the decay behaviour was very different to that observed for NsrR, with the [4Fe–4S] WhiD peak decaying gradually throughout the titration, being lost entirely only at [NO] : [4Fe–4S] ≥ 10; consistent with earlier spectroscopic studies.^86^ Cluster nitrosylation peaks were observed at +30 Da, +60 Da in WhiD spectra and persisted over a broad range of NO concentrations until the [4Fe–4S] WhiD peak decayed away. Isotope shift analysis (natural abundance *vs.*^57^Fe/^34^S enriched cluster) revealed a mass difference of +12 Da, consistent with their assignment as [4Fe–4S](NO) and [4Fe–4S](NO)_2_, respectively (Fig. 6b).^47^ A further peak at +120 Da, corresponding to a tetranitrosylated cluster, [4Fe–4S](NO)_4_, was also observed.^47,94^ At higher ratios of NO, RBS-type species were detected, see Fig. 6c.^47^

Native MS has also been used to investigate the interaction of Wbl proteins with the principle Sigma factor, an important part of the cellular transcriptional machinery. Native MS studies of both WhiB1 from *M. tuberculosis* and WhiD from *S. venezuelae* have demonstrated 1 : 1 complex formation that is dependent on the [4Fe–4S] cluster. These complexes were either insensitive (WhiB1) or less sensitive (WhiD) to O_2_, but reacted rapidly with NO, leading to dissociation of the complex,^46,103^ supporting the idea that these proteins function in sensing NO.

## 5. Conclusions

Native MS is a powerful methodology that can yield unprecedented detail of the chemistry taking place at the active sites of proteins, and in doing so provides unusually clear insight into protein function. The work highlighted here demonstrates the application of native MS in studies of more fragile (relative to ferredoxins) Fe–S proteins, and the feasibility of time-resolved native MS studies of reactions of Fe–S cluster cofactors within a protein framework. Isotope substitution data, from ^34^S-, ^57^Fe- or ^34^S/^57^Fe-substituted forms of Fe–S proteins, are crucial for providing and unambiguous confirmation of peak assignments for cluster conversion intermediates and products, allowing differentiation between them and naturally occurring background adducts.^44,47,48,104,105^ The wealth of mechanistic data obtainable from native MS suggests that it is likely to find broad application in studies of wider range of protein cofactors systems involving interactions/reactions that result in changes in mass.

## Conflicts of interest

There are no conflicts to declare.
